# Real and synthetic Punjabi speech datasets for automatic speech recognition

**DOI:** 10.1016/j.dib.2023.109865

**Published:** 2023-11-27

**Authors:** Satwinder Singh, Feng Hou, Ruili Wang

**Affiliations:** School of Mathematical and Computational Sciences, Massey University, Auckland, New Zealand

**Keywords:** Automatic speech recognition, low-resource languages, Speech dataset, Punjabi language

## Abstract

Automatic speech recognition (ASR) has been an active area of research. Training with large annotated datasets is the key to the development of robust ASR systems. However, most available datasets are focused on high-resource languages like English, leaving a significant gap for low-resource languages. Among these languages is Punjabi, despite its large number of speakers, Punjabi lacks high-quality annotated datasets for accurate speech recognition. To address this gap, we introduce three labeled Punjabi speech datasets: Punjabi Speech (real speech dataset) and Google-synth/CMU-synth (synthesized speech datasets). The Punjabi Speech dataset consists of read speech recordings captured in various environments, including both studio and open settings. In addition, the Google-synth dataset is synthesized using Google's Punjabi text-to-speech cloud services. Furthermore, the CMU-synth dataset is created using the Clustergen model available in the Festival speech synthesis system developed by CMU. These datasets aim to facilitate the development of accurate Punjabi speech recognition systems, bridging the resource gap for this important language.

Specifications Table

**Table:** 

Subject	Computer science, Signal processing
Specific subject area	Automatic speech recognition and Synthesis
Data format	Raw Digital audio files (WAV files) and their corresponding text transcriptions (TSV files).
Type of data	Audio and text
Data collection	The Punjabi Speech dataset is compiled by recording text sourced from an Old Newspaper corpus. Additionally, synthesized datasets are generated using Google's text-to-speech (TTS) service (Google-synth) and CMU's Clustergen TTS model (CMU-synth). We filter Punjabi text in Old Newspaper corpus and preprocess it to remove special symbols and numerical characters.The following tools are used in the process: • Smartphones, iPad, Rode USB, and Rode NT-2 studio mic for recording the audio. • Google's TTS services and the Festival Speech Synthesis system for synthesizing audio. • Audacity software for audio processing. • Windows and Linux operating systems.
Data source location	• Institution: Massey University • City/Town/Region: Auckland • Country: New Zealand Primary data source location: • Old Newspapers text corpus: https://www.kaggle.com/datasets/alvations/old-newspapers • HC Corpora newspapers: https://www.kaggle.com/code/mpwolke/hc-corpora-newspapers/notebook
Data accessibility	Punjabi Speech datasetRepository name: Mendeley DataData identification number: 10.17632/sdbc8f5b77.2 Direct URL to data: https://data.mendeley.com/datasets/sdbc8f5b77/2 Google-synth and CMU-synth datasetsRepository name: FigshareData identification number: • Google-synth: 10.6084/m9.figshare.23615607.v1 • CMU-synth: 10.6084/m9.figshare.23606697.v1 Direct URL to data: • Google-synth: https://figshare.com/articles/dataset/Google-synth_A_Synthesized_Punjabi_Speech_Dataset/23615607 • CMU-synth: https://figshare.com/articles/dataset/_strong_CMU-synth_A_synthesized_Punjabi_Speech_dataset_strong_/23606697
Related research article	Satwinder Singh, Feng Hou, and Ruili Wang. 2023. A Novel Self-training Approach for Low-resource Speech Recognition. Proc. INTERSPEECH, 1588-1592 DOI: https://doi.org/10.21437/Interspeech.2023-540

## 1 Value of the Data

• Speech data is important for Punjabi speech recognition as it provides the necessary foundation for training accurate speech recognition systems specific to the Punjabi language.
• Punjabi is considered a low-resource language, lacking high-quality annotated datasets required for building robust speech recognition systems. The creation of Punjabi speech datasets, including real-speech and synthesized datasets, helps address this scarcity and enables the development of accurate Punjabi ASR systems.
• Researchers, Punjabi speakers, and developers benefit from these datasets by improving transcription services, aiding linguistic research, and facilitating advancements in Punjabi language technology.

## 2 Data Description

Automatic speech recognition has been an active area of research for several decades, and the availability of large annotated datasets has played a crucial role in the development of robust speech recognition systems [1,2]. In recent years, with the advent of deep learning and other machine learning techniques, there has been a renewed interest in creating and using large datasets to train speech recognition models.

There are many speech datasets available such as Common Voice [3], LibriSpeech [4], TIMIT [5], TED-LIUM [6], Wall Street Journal [7], Switchboard [8], Google Speech Commands datasets [9] and so on. However, most of these datasets are compiled for high-resource languages such as English. Most of the languages of the world are low-resource languages and do not have enough linguistic resources such as high-quality annotated datasets. There are approximately 7000 languages spoken worldwide, but only a small fraction of them, roughly 100, have well-established automatic speech recognition (ASR) systems [10]. The remaining languages, including Punjabi, are considered low-resource languages. Punjabi, belonging to the Indo-Aryan language family, is spoken by over 110 million native speakers in India and Pakistan, as well as throughout the world. Punjabi is unique in the Indo-Aryan language family because it uses distinct lexical tones, including low, mid, and high tones, and is written in two scripts: Gurmukhi in India and Shahmukhi in Pakistan. Despite its large population of speakers, Punjabi lacks the quality annotated datasets to build an accurate speech recognition system.

There are two primary datasets available for the Punjabi language: Common Voice [3] and Shrutilipi [11]. Common Voice is a crowdsourced dataset encompassing various languages, offering diversity in speakers. However, it often suffers from audio quality disparities, background noises, and variable recording devices, impacting the accuracy of speech recognition systems. Additionally, the Shrutilipi dataset comprises paired audio and text data sourced from public platforms like All India Radio news bulletins, obtained through data mining techniques. This dataset covers 12 distinct Indian languages, including Punjabi. Nevertheless, it faces challenges related to misalignment and labeling accuracy.

Further, numerous studies have explored the utilization of speech synthesis data to enhance automatic speech recognition (ASR) performance. Combining real and synthetic speech generated by models like Tacotron-2 has shown improved results for the ASR systems [12]. Moreover, enhancing diversity through multi-speaker speech synthesis has further boosted ASR performance [13]. Additionally, Tjandra et al. achieved promising outcomes through joint training of ASR and TTS systems in their SpeechChain model [14]. Furthermore, Chen et al. introduced the tts4pretrain system, leveraging text to incorporate valuable phonetic and lexical knowledge during the pre-training stage [15]. The subsequent development, tts4pretrain 2.0, incorporated consistency regularization and contrastive loss during pretraining, enhancing the learning of a robust shared representation of speech and text [16].

With that in mind, we create three labeled Punjabi speech datasets namely, Punjabi Speech [17], Google-synth [18], and CMU-synth [19]. In our data collection process of Punjabi Speech dataset, akin to Common Voice, we maintain a controlled recording environment. Our audios are captured in both studio settings using high-quality microphones and on smartphones, incorporating natural background noise. This meticulous approach allows us greater control over audio quality and diversity in our real Punjabi Speech dataset. Additionally, our synthesized datasets (i.e., Google-synth and CMU-synth) serve as valuable resources to further enhance ASR performance.

### 2.1 Punjabi Speech Dataset

**Fig. 1:**
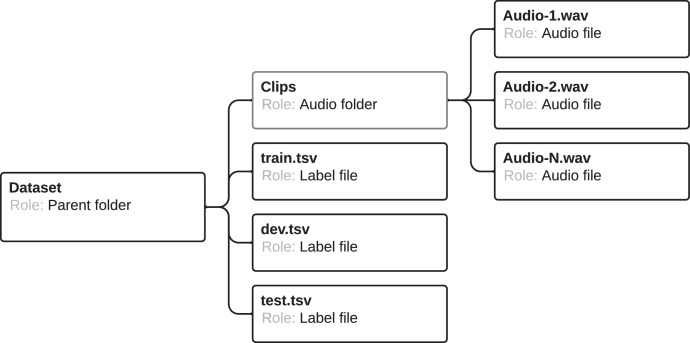
Directory structure of our datasets. The Google-synth and CMU-synth follow the same directory structure as Punjabi Speech dataset, except the audio file name only includes UtteranceID.

The Punjabi Speech dataset is a read speech dataset, recorded in the studio and open environment. Presently, this dataset contains speech samples from two male speakers and has a total of 2429 spoken utterances making it 4 h of data. We pre-define the data splits with 80 % for training and 10 % for validation and 10 % for testing purposes. The Punjabi speech dataset follows a very simple structure. All the speech files are present in clips directory and all the transcript files (train, dev, test) in TSV format are present in the parent directory of the corpus as illustrated in Fig. 1.

In transcript files, each line represents a label for a single speech sample present in the *clips* directory. The first column in the line represents the path/name to the WAV file, and the second column separated by a tab holds the actual transcript in text form as illustrated in Table 1 and the following figure.

**Table 1:** Few samples from the Punjabi Speech dataset. Note that the TSV files do not have translated ground truth labels. This is added to make it more readable.



**Fig. 2:**
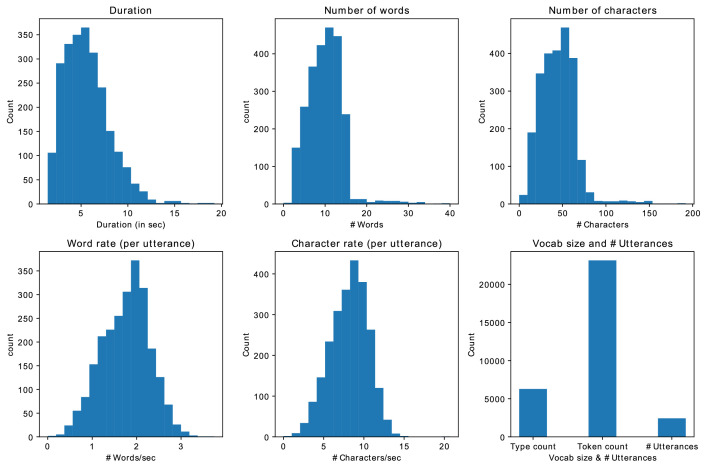
Statistics of Punjabi Speech dataset

Fig. 2 demonstrates that most audio recordings fall within the 2 to 15 s range, with an average duration of 5 to 7 s. On average, these recordings contain approximately 10 words and 45 characters and are spoken at a rate of 0.5 to 3 words per second. The Punjabi Speech dataset comprises 6281 unique words, with a total word count of 23,134 tokens.

### 2.2 Google-Synth Dataset

**Fig. 3:**
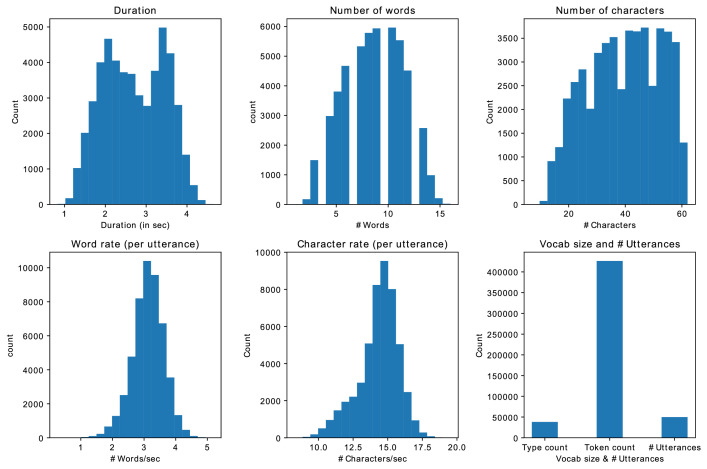
Statistics of Google-synth dataset

Fig. 3 demonstrates the dataset statistics for the Google-synth dataset. Most of the utterances in the dataset are between 2 and 4 s in duration, with a total range spanning from 1 to 4 s. On average, each utterance contains approximately 8 words. The rate of speech is estimated to be roughly 3 words per second or 15 characters per second. The dataset contains a vocabulary of 38,281 words and a token count of 426,317.

### 2.3 CMU-Synth Dataset

**Fig. 4:**
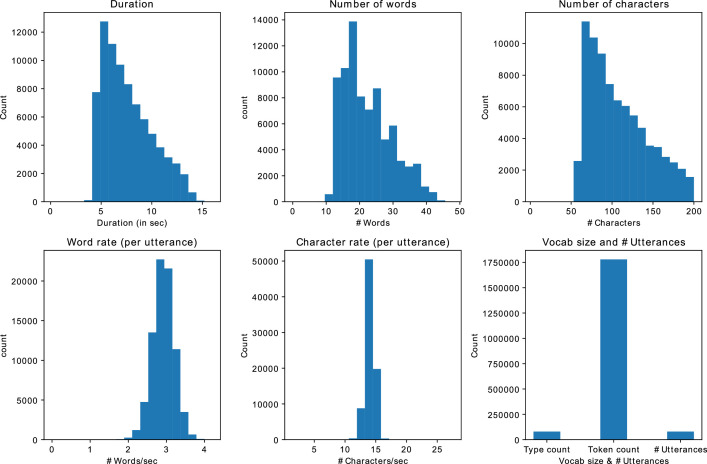
Statistics of CMU-synth dataset

We generated around 80 K utterances, which equals to 170 h of speech data. As shown in Fig. 4, on average, each utterance contains approximately 108 characters/ 22 words. The rate of speech is roughly around 3 words per second or 14 characters per second. The dataset contains a vocabulary of 38,281 words and a token count of 426,317.

## 3 Overview of Vocabulary Overlap Between Datasets

The analysis of dataset overlaps reveals significant linguistic commonalities and distinctions among the Punjabi Speech, Google-synth, and CMU-synth datasets.

• Punjabi Speech and Google-synth: A 91.18 % overlap is observed between the Punjabi Speech and Google-synth datasets, suggesting a substantial shared vocabulary and linguistic resemblance.
• Google-synth and CMU-synth: The Google-synth and CMU-synth datasets exhibit a moderate overlap of 70.03 %, indicating some linguistic similarities while maintaining distinct elements.
• Punjabi Speech and CMU-synth: Notably, the Punjabi Speech and CMU-synth datasets showcase a 92.42 % overlap, emphasizing a significant convergence of language elements between these two datasets.

## 4 Experimental Design, Materials and Methods

**Fig. 5:**
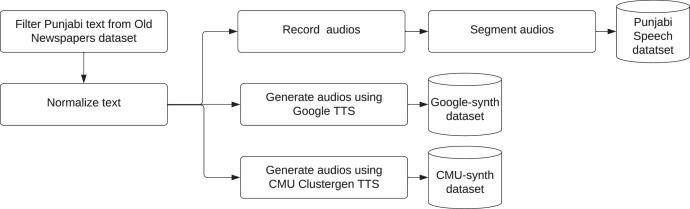
Datasets production flow.

Fig. 5 shows the dataset production flow. For recording a Punjabi Speech and synthesizing Google-synth and CMU-synth datasets, we utilize text available in the Old Newspapers dataset^1^. This dataset is a carefully curated subset of the HC corpus^2^, and it is available to the public for free under the CC0 public domain license. The corpus contains a vast amount of textual data that has been collected from a wide range of sources, including newspapers, blogs, and various social media platforms. This corpus has been designed to cover 67 different languages spoken across the world, and it comprises 16,806,041 sentences in the TSV (Tab Separated Values) file format.

As our focus is on the Punjabi language, we filtered out the Punjabi sentences from the original corpus. This leaves us with a more manageable dataset that we can work with easily. To normalize the text, we filter out all the sentences containing special symbols and numeric entries.

The Punjabi Speech dataset is a read speech dataset, recorded in the studio and open environment. We record the speech samples at 44,100 Hz in WAV file format. In the studio, we utilize Rode NT-USB and Rode NT2A Studio Microphones record using Audacity software. In open environment, we recorded the audios using smart phone and iPad 11 Pro device microphones. We keep our recording below 15 s to avoid memory issues while training on the GPUs.

For Google-synth, we used Google Text-to-Speech Cloud API^3^, which supports a wide range of languages and voices, and users can customize the speech rate, pitch, and volume to suit their preferences. For Punjabi (language code="pa-Guru-IN"), Google offers TTS models in four different voices (2 male and 2 female). We carefully selected around 50,000 sentences from Old Newspapers dataset for synthesis. With 50,000 utterances, we generated about 38 h of speech. We synthesize speech at 44,100 Hz with a similar directory style as the Punjabi Speech dataset.

Further, we produce a CMU-synth dataset using CMU's Clustergen TTS model [20]. Clustergen is a statistical parametric model that is incorporated within the Festival Speech Synthesis system. We train the Clustergen TTS model from scratch using CMU INDIC Punjabi dataset^4^, which comprises 0.4 h of annotated speech data from a single female speaker. In total, we produced approximately 80,000 utterances, which corresponds to roughly 170 h of synthesized audio.

## Limitations

Not applicable.

## Ethics Statement

Informed consent was obtained from all subjects involved in the audio recording process.

## CRediT authorship contribution statement

**Satwinder Singh:** Writing – review & editing, Data curation, Conceptualization, Investigation, Methodology, Project administration, Software, Validation. **Feng Hou:** Supervision, Writing – review & editing, Conceptualization, Investigation. **Ruili Wang:** Supervision, Writing – review & editing, Investigation.

## Data Availability

Google-synth: A Synthesized Punjabi Speech Dataset (Original data) (Figshare) Punjabi Speech: A labeled Speech Corpus (Original data) (Mendeley Data) CMU-synth: A synthesized Punjabi Speech dataset (Original data) (Figshare)

## Footnotes

1 https://www.kaggle.com/alvations/old-newspapers

2 https://www.kaggle.com/code/mpwolke/hc-corpora-newspapers/notebook

3 https://cloud.google.com/text-to-speech

4 http://festvox.org/cmu_indic
